# Synthesis, Characterization and Antimicrobial Activity of d^8-10^ Metal Complexes of some 2-Substituted-1H-Benzimidazoles

**DOI:** 10.1155/MBD.1999.163

**Published:** 1999

**Authors:** Bahri Ülküseven, Aydin Tavman, Gülten Ötük

**Affiliations:** 1 Istanbul University Faculty of Engineering Science Department of Chemistry Avcılar Istanbul Turkey; 2 Istanbul University Faculty of Pharmacy Department of Microbiology Beyazid Istanbul Turkey

## Abstract

The metal complexes of nine 2-substituted-1H-benzimidazoles **(I-IX)** with Ni(II), Pd(II), Cu(II), Ag(I), Zn(II) salts were synthesized. The compounds were characterized by melting point, analytical data, IR spectroscopy and magnetic susceptibility. The antimicrobial activity of the compounds was determined by the disk diffusion method in Mueller-Hinton Agar on *Staphylococcus aureus* *ATCC 6538*, *Staphylococcus epidermidis ATCC 12228*, *Escherichia coli ATCC 8739*, *Klebsiella pneumoniae ATCC 4352*, *Pseudomonas aeruginosa ATCC 1539*, *Salmonella typhi*, *Shigella flexneri*, *Proteus mirabilis*, *Candida albicans ATCC 10231*. Cu(II)and Ag(I)complexes of **II, III ** and **IV** showed considerable activity against *S. aureus*, *S. epidermidis*, *Ps. aeruginosa*, *S. typhi*, *Sh. flexneri* and *C. albicans* microorganisms, the ligands themselves having no effect.


### SYNTHESIS, CHARACTERIZATION AND ANTIMICROBIAL ACTIVITY OF $d^{8-10}$ METAL COMPLEXES OF SOME 2-SUBSTITUTED-1H-BENZIMIDAZOLES

Bahri Ülküseven*[1], Aydın Tavman[1] and Gülten Ötük[2]

[1] Istanbul University, Faculty of Engineering Science, Department of Chemistry, Avcılar, Istanbul, Turkey
[2] Istanbul University, Faculty of Pharmacy, Department of Microbiology, Beyazıd, Istanbul, Turkey

### ABSTRACT

The metal complexes of nine 2-substituted-1H-benzimidazoles (**I-IX**) with Ni(II), Pd(II), Cu(II), Ag(I), Zn(II) salts were synthesized. The compounds were characterized by melting point, analytical data, IR spectroscopy and magnetic susceptibility. The antimicrobial activity of the compounds was determined by the disk diffusion method in Mueller-Hinton Agar on *Staphylococcus aureus ATCC 6538, Staphylococcus epidermidis ATCC 12228, Escherichia coli ATCC 8739, Klebsiella pneumoniae ATCC 4352, Pseudomonas aeruginosa ATCC 1539, Salmonella typhi, Shigella flexneri, Proteus mirabilis, Candida albicans ATCC 10231.* Cu(II) and Ag(I) complexes of **II**, **III** and **IV** showed considerable activity against *S. aureus*, *S. epidermidis*, *Ps. aeruginosa*, *S. typhi*, *Sh. flexneri* and *C. albicans* microorganisms, the ligands themselves having no effect.

### INTRODUCTION

2-Substituted-1H-benzimidazoles have been associated with various types of biological effects such as analgesic, antelmintic, antispasmodic, antifungal, antiviral and antiulcer activities[1–7]. In spite of the fact that there are innumerable studies in this matter, the investigation concerned with biological activities of metal complexes of 2-substituted-1H-benzimidazoles has been limited. Srivastava investigated metal chelates of 2-(2'-hydroxybenzylidene)-aminobenzimidazole and compared the antifungal activity of the ligand with its metal chelates[8]. In another study, it has been observed that the metal complexes were more fungitoxic than corresponding ligand[9].

Nine 2-substituted-1H-benzimidazole derivatives (**I-IX**) (Figures 1 and 2) and their 74 complexes formed by reactions with $NiCl_2$, $PdCl_2$, $Pd(NO_3)_2$, $CuCl_2$, $Cu(CH_3COO)_2$, $Cu(ClO_4)_2$, $AgNO_3$, $ZnCl_2$, $ZnSO_4$ have been isolated in pure form for first time and characterized by using analytical data and IR spectroscopy. The compounds **I-IV**, **VIII** and **IX** have variously biological activities, which are antinematodic[10], fungicide[11], antelmintic[12], sedative[13] and antipoliovirus[14] for **I**, antifungal[15], antinematodic[10,16], antelmintic[17], tuberculostatic[18] and antiinflammatory[19] for **II**, anti-inflammatory[20] for **III**, antifungal[15], nematocide[21], tuberculostatic[18] and antelmintic[17,22] for **IV**, antipoliovirus[23] and antifungal[24] for **VIII**. The compound **IX** decreased the yield of intracellular virus[25]. However, no biological potential of **V**, **VI** and **VII** was found until now.

In this research, the microbiological activity of the complexes were examined and compared with that of the ligands by means of the disk diffusion method.

**Figure 1**

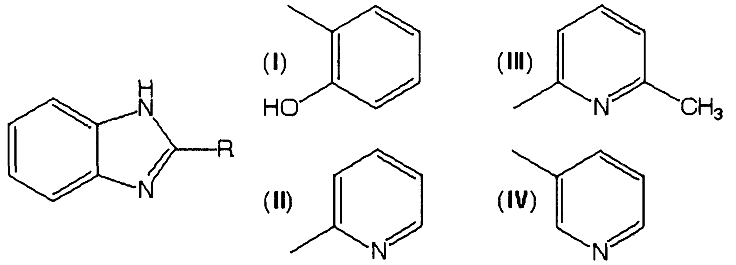

**Figure 2**

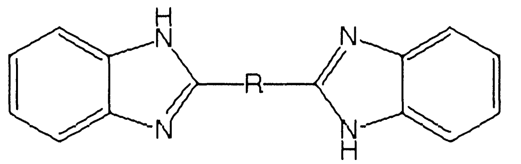

(**V**) $-CH_2-CH_2-$

(**VI**) $-CH=CH-$

(**VII**) $-CH_2-CH_2-CH_2-$

(**VIII**) $-CHOH-CHOH-$

(**IX**) $-(CHOH)_4-$

**Table 1:** Analytical data and some physical properties of the metal complexes, exhibiting some antimicrobial activity, of **I-IV**, **VI** and **VIII**.

| Compound | Color | Mp °C | Yield (%) | Elemental Analysis Found (Calculated) %C | %H | %N | %M | %Cl | $\mu_{eff}$ | Solubility |
|---|---|---|---|---|---|---|---|---|---|---|
| $C_{13}H_{10}N_2O$<br>**I** | cls. | 243 | 75 | 74.33<br>(74.28) | 4.81<br>(4.76) | 13.39<br>(13.33) | | | – | EtOH<br>DMSO,AcH |
| Ag[**I**]$_2NO_3$<br>$C_{26}H_{20}AgN_5O_5$ | light pink | 232 | 70 | 51.67<br>(52.88) | 3.50<br>(3.39) | 9.68<br>(9.49) | 29.07<br>(28.39) | | 0 | DMSO<br>AcH |
| **II**<br>$C_{12}H_9N_3$ | cls. | 218 | 40 | 73.96<br>(73.84) | 4.78<br>(4.61) | 21.38<br>(21.54) | | | – | EtOH<br>DMSO,AcH |
| Ag[**II**]$NO_3$<br>$C_{12}H_9AgN_4O_3$ | Light brown | >350 | 70 | 40.46<br>(39.45) | 2.45<br>(2.46) | 14.81<br>(15.34) | 30.11<br>(29.57) | | 0 | DMSO<br>AcH |
| Cu[**II**]$_2Cl_2$<br>$C_{24}H_{18}Cl_2CuN_6$ | Green | 330 | 70 | 54.82<br>(54.91) | 3.38<br>(3.43) | 15.95<br>(16.01) | 12.46<br>(12.11) | 21.20<br>(21.55) | 1.7<br>2 | DMSO<br>AcH |
| Cu[**II**]$_2(ClO_4)_2$<br>$C_{24}H_{18}Cl_2CuN_6O_8$ | blue-green | 205 | 60 | 44.21<br>(44.14) | 2.85<br>(2.76) | 12.85<br>(12.87) | 8.88<br>(9.74) | <br>(10.88) | 1.5<br>0 | DMSO |
| **III**<br>$C_{13}H_{11}N_3$ | cls. | 222 | 35 | 74.67<br>(74.64) | 5.29<br>(5.26) | 20.04<br>(20.09) | | | 0 | EtOH<br>DMSO |
| Ag[**III**]$NO_3$<br>$C_{13}H_{11}AgN_4O_3$ | Light pink | 243 | 70 | 41.09<br>(41.16) | 2.25<br>(2.90) | 13.79<br>(14.77) | 28.86<br>(28.47) | | 0 | DMSO<br>AcH |
| Cu[**III**]$Cl_2$<br>$C_{13}H_{11}Cl_2CuN_3$ | dark yellow | 278 | 70 | 45.43<br>(45.41) | 3.26<br>(3.20) | 11.82<br>(12.22) | 19.10<br>(18.49) | 20.16<br>(20.67) | 1.6<br>8 | DMSO |
| Cu[**III**]$_2Cl_2$<br>$C_{26}H_{22}Cl_2CuN_6$ | yellow | 285 | 65 | 56.44<br>(56.47) | 3.95<br>(3.98) | 15.08<br>(15.20) | 11.77<br>(11.50) | 12.19<br>(12.85) | 1.7<br>5 | DMSO<br>EtOH |
| Cu[**III**]$_2(AcO)_2$<br>$C_{30}H_{28}CuN_6O_4$ | light green | 206 | 70 | 59.10<br>(60.05) | 4.42<br>(4.67) | 13.61<br>(14.01) | 9.71<br>(10.60) | | 1.7<br>1 | DMSO<br>AcH |
| Cu[**III**]$_2(ClO_4)_2$<br>$C_{26}H_{22}Cl_2CuN_6O_8$ | light brown | 262 | 65 | 45.52<br>(45.85) | 3.24<br>(3.23) | 12.05<br>(12.34) | 9.93<br>(9.34) | <br>(10.43) | 1.7<br>4 | DMSO,<br>EtOH |
| Ag[**IV**]$NO_3$<br>$C_{12}H_9AgN_4O_3$ | cls. | 301 | 75 | 39.28<br>(39.45) | 2.49<br>(2.46) | 14.92<br>(15.34) | 29.75<br>(29.56) | | 0 | DMSO<br>AcH |
| Cu[**VI**]$Cl_2$<br>$C_{16}H_{12}Cl_2CuN_4$ | olive green | 315 | 60 | 48.76<br>(48.66) | 3.11<br>(3.04) | 14.09<br>(14.19) | 15.63<br>(16.10) | 17.76<br>(17.99) | 1.3<br>7 | DMSO<br>AcH |
| Cu[**VIII**]$(ClO_4)_2$<br>$C_{16}H_{13}Cl_2CuN_4O_{10}$ | blue | 216 | 75 | 34.37<br>(34.52) | 2.62<br>(2.53) | 10.45<br>(10.06) | 11.72<br>(11.41) | <br>(12.74) | 1.0<br>9 | DMSO, AcH<br>water, EtOH |

M.p.: melting point. $\mu_{eff}$: magnetic moment; measured in DMSO, $\Omega^{-1}cm^2mol^{-1}$, at 25±1°C. cls.: colorless.

### MATERIALS AND METHODS

### Preparation of the ligands

The ligands **I-IV**, were prepared by modifying slightly the literature method[1] and **V-IX** according to the Phillips method[26]. Only ligand **VI** was obtained by using the melting process [27].

Aldehydes used to synthesize ligands **I-IV** and the yields obtained are: salicylaldehyde, 85% (**I**); pyridine-2-carboxaldehyde, 37% (**II**); 6-methyl-pyridine-2-carboxaldehyde, 35% (**III**); pyridine-3-carboxaldehyde, 55% (**IV**), respectively.

The dicarboxylic acids used and the yields obtained are: **V**, succinic acid, 78%; **VI**, maleic acid, 20%; **VII**, glutaric acid, 77%; **VIII**, tartaric acid, 75%; **IX**, mucic acid, 62%, respectively.

**Preparation of metal complexes in ethanol/water**

A 5 mL aqueous solution of 0.04 M metal salts (0.2 mmole; e.g. 50 mg $NiCl_2 \cdot 6H_2O$) and 0.4 mmole ligand (e.g. 80 mg **II**) in 25 mL of ethanol in a reaction tube was stirred vigorously. The solution mixture was refluxed with stirring for about 4 hours. The metal complexes with $AgNO_3$ were obtained for one hour at 50±5°C. The mixture was then allowed to stand at room temperature overnight to give a solid product. This was then filtered, washed with water and ethanol, dried under *vacuo* over anhydrous $CaCl_2$.

Compositions and purity of all the ligands and metal complexes were checked by using microanalysis, IR, TLC and melting points.

**Table 2**: Some selected IR bands of the metal complexes showed antimicrobial activity, of **I-IV**, **VI** and **VIII**. ($cm^{-1}$, in KBr disc)[a].

| Compound | ν(NH) | $\nu(C=N)_{im}$ | $\nu(C=N)_{py}$ | δ(NH) | Anion |
|---|---|---|---|---|---|
| Ligand I[b] | 3230 | 1600 | – | 1538 | – |
| $Ag[I]_2NO_3$ | 3246 | 1608 | – | 1538 | 1384 |
| Ligand II | H-bond. | 1592 | 1569 | 1546 | – |
| $Cu[II]_2Cl_2$ | 3423 | 1615 | 1608 | 1546 | – |
| $Cu[II]_2(ClO_4)_2$ | 3438 | 1638 | 1608 | 1577 | 1100 |
| $Ag[II]NO_3$ | 3431 | 1638 | 1600 | 1569 | 1384 |
| Ligand III | H-bond. | 1600 | 1577 | 1538 | – |
| $Cu[III]Cl_2$ | 3169 | 1615 | 1584 | 1546 | – |
| $Cu[III]_2Cl_2$ | 3107 | 1615 | 1592 | 1546 | – |
| $Cu[III]_2(Ac)_2$ | 3423 | 1608 | 1577 | 1546 | 1415 |
| $Cu[III]_2(ClO_4)_2$ | 3431 | 1615 | 1584 | 1546 | 1092 |
| $Ag[III]NO_3$ | 3192 | 1600 | 1577 | 1538 | 1384 |
| Ligand IV | H-bond. | 1600 | 1584 | 1546 | – |
| $Ag[IV]NO_3$ | 3231 | 1631 | 1600 | 1546 | 1384 |
| Ligand VI[b] | H-bond. | 1592 | – | 1546 | – |
| $Cu[VI]Cl_2$ | 3431 | 1577 | – | 1523 | – |
| Ligand VIII[b] | H-bond. | 1592 | – | 1538 | – |
| $Cu[VIII](ClO_4)_2$ | 3223 | 1608 | – | 1531 | ~1100 |

[a] $\nu(C=N)_{im}$ and $\nu(C=N)_{py}$ bands medium, the ν(NH) and ν(OH) broad, ν(C–O) bands sharp and δ(NH) bands weak.

[b] Frequencies of $\nu(C=C)_{al.}$ and $\nu(C=C)_{ar}$ are 1623 and 1631, respectively. The streching frequencies of phenolic C–O are between 1269-1284 $cm^{-1}$ and of alcoholic C–O groups between 1061-1160 $cm^{-1}$. ν(OH) bands appear in 3320-3400 $cm^{-1}$ range.

**Antimicrobial activity of the compounds**

The disk diffusion method was used for determining the antimicrobial activity. The antibacterial activity against *Staphylococcus aureus ATCC 6538, S. epidermidis ATCC 12228, Escherichia coli ATCC 8739, Klebsiella pneumoniae ATCC 4352, Pseudomonas aeruginosa ATCC 1539, Salmonella typhi, Shigella flexneri* and the antifungal activity against *Candida albicans ATCC 10231* were investigated. Mueller-Hinton agar (Difco, Detroit, USA) was melted at 100°C and then cooled to 56°C, was poured into plates of 9 cm diameter in quantities of 20 mL, and left on a flat surface to solidify and the surface of the medium was dried at 37°C. Then, cultures of each bacteria and yeast strain, after being kept in Mueller-Hinton broth (Difco) at 37°C for 18-24 hours and diluted with Mueller-Hinton broth to $10^5$ cfu/mL, were pipetted into the Mueller-Hinton agar plates prepared as described above. The surface of the medium was allowed to dry. The 10 mg/mL (in DMSO, E. Merck) compound impregnated disks were applied to the surface of inoculated plates. The plates were placed in an incubator at 37°C. After 18-24 hours of incubation, the plates were examined and the compounds which were found effective against the strains were selected in order to determine the minimum inhibitory concentrations.

*Determination of MIC* The minimum inhibitory concentrations were determined by the microbroth dilution technique using Mueller-Hinton broth. Serial two-fold dilutions ranging from 5000 to 4.8 mcg/mL were prepared in Mueller-Hinton broth. The inoculum was prepared with a 4-6 hours broth culture of each strain adjusted to a turbidity equivalen to 0.5 McFarland standard, diluted in Mueller-Hinton broth to give a final concentration of $5x10^8$ cfu/L in the test tray. The trays were covered and placed in plastic bags to prevent drying; incubation was at 37°C for 18-20 hours. The MIC was defined as the lowest concentration of compound giving complete inhibition of visible growth. MIC values of compounds are given Table 3.

**Table 3**: MIC values (mcg/mL) of the compounds

| Compound | Microorganism [a] | | | | | | | |
|---|---|---|---|---|---|---|---|---|
| | A | B | C | D | E | F | G | H |
| Ag[**I**]$_2$NO$_3$ | 39 | 39 | 39 | 39 | 19.5 | 39 | 39 | 39 |
| **II** | – | – | – | – | – | – | – | 250 |
| Ag[**II**]NO$_3$ | 9.8 | 19.5 | 19.5 | 19.5 | 19.5 | 19.5 | 19.5 | 19.5 |
| Cu[**II**]$_2$Cl$_2$ | 78 | 39 | – | – | – | – | – | – |
| Cu[**II**]$_2$(ClO$_4$)$_2$ | 78 | 78 | – | – | – | – | 78 | – |
| **III** | – | – | – | – | – | – | – | 250 |
| Ag[**III**]NO$_3$ | 9.8 | 9.8 | 9.8 | 39 | 9.8 | 9.8 | 9.8 | 9.8 |
| Cu[**III**]Cl$_2$ | 156 | 78 | – | – | – | – | – | 250 |
| Cu[**III**]$_2$Cl$_2$ | 156 | 78 | – | – | – | – | – | 156 |
| Cu[**III**]$_2$(Ac)$_2$ | 250 | 125 | – | – | – | – | – | – |
| Cu[**III**]$_2$(ClO$_4$)$_2$ | 156 | 78 | – | – | – | – | – | – |
| Ag[**IV**]NO$_3$ | 19.5 | 19.5 | 19.5 | 19.5 | 19.5 | 19.5 | 19.5 | 19.5 |
| Cu[**VI**]Cl$_2$ | – | 39 | – | – | – | – | – | – |
| Cu[**VIII**](ClO$_4$)$_2$ | – | 9.8 | – | – | – | – | – | – |
| AgNO$_3$ | 9.8 | 4.9 | 4.9 | 4.9 | 4.9 | – | – | 4.9 |

[a] : A, *Staphylococcus aureus ATCC 6538;* B, *Staphylococcus epidermidis ATCC 12228;* C, *Escherichia coli ATCC 8739;* D, *Klebsiella pneumoniae ATCC 4352;* E, *Pseudomonas aeruginosa ATCC 1539;* F, *Salmonella typhi;* G, *Shigella flexneri;* H, *Candida albicans ATCC 10231.*

– : very low MIC value.

### RESULTS AND DISCUSSIONS

### Composition of the compounds

The interaction of the ligands (**I-IX**) with the metal salts in a 1:2 molar ratio in ethanol yielded stable solid complexes corresponding to the general formulas [M(L)A$_n$] and [M(L)$_2$A$_n$] (where A = AcO$^-$, Cl$^-$, ClO$_4^-$ and NO$_3^-$. *n* equals 1 in the all of Ag(I) complexes, and 2 in the other complexes). All the complexes are very poorly soluble in common organic solvents such as dichloromethane and polar solvents such as EtOH and MeOH, but are moderate soluble in donor solvents, such as dimethylsulfoxide (DMSO). The analytical results and some physical properties of these compounds are shown in Table 1.

Some selected IR bands of the complexes are shown in Table 2. According to literature, the coordinations occur through the C=N nitrogen atom of imidazole in all the complexes[28]. The ν(C=N) bands of imidazole and the ν(C=N) bands of pyridine of the ligands **II**, **III** and **IV** can be clearly seen in the IR spectra. Imidazole ν(C=N) band in all the metal complexes and both ν(C=N) bands in the metal complexes of **II-IV** are shifted to higher frequencies compared with those of the free ligands by 5–40 $cm^{-1}$. Similarly, the ν(C–O) band frequencies of phenol and alcohol groups in the ligands increase on complexation. In addition to this, the characteristic acetate, nitrate and perchlorate bands confirm the suggested tentative formula of the complexes.

### Antimicrobial activity of the ligands and their metal complexes

The antimicrobial activity (MIC, μg/mL) of the compounds are shown in Table 3. From Table 3, the complexes of **III** with Cu(II) and Ag(I) have various antimicrobial activities. Cu(II) salts and **II** and **III** ligands which are not effective alone to two (*S. aureus* and *S. epideridis*) of the nine bacteria, however, they show some effect in their Cu(II)-complex form. This effect can be logically explained by the fact that the benzimidazole derivatives must be activated by Cu(II) ions in some way (maybe synergetic activity). However, we think that the activity was revealed as a result of the independent characteristic of the metal complexes. We presume that this effect is due to the formation of an "enzyme-Cu(II)-benzimidazole" mixed complex, by the bonding of metal complexes through the metal atom to the microorganism's cell enzymes. The ternary complexes, composed of enzyme, metal ion and organic ligand, may be likely proposed considering the results in the some biological studies[29-32]. The benzimidazole derivative, which is the organic portion of this complex structure, is responsible for the ineffectiveness of the said Cu(II) complexes to the other bacteria except *S. aureus* and *S. epidermidis*. The expected activity, in the form of Cu(II) complexes, will be obtained by appropriate benzimidazole derivatives, even though they may not be active alone by themselves.

**Acknowledgments:** This work was supported by the Research Fund of The University of Istanbul. Project number: 677/301194.

**REFERENCES**

1 Yıldır, I., Uzbay, T., Noyanalpan, N., *J. Fac. Pharm. Gazi,* **7(2)**, 111-24 (1990).
2 Kline, S., French Laboratories, *Brit.,* 1,122,957 (1965).
3 Öztürk, Y., Isıkdag, I., *FABAD,* **13(1)**, 326-31 (1988).
4 Öztürk, Y., Isıkdag, I., Uçucu, Ü., Demirayak, S., *FABAD,* **13**, 326-31 (1988); Bahadur, S., Pandey, K. K., *Pharmazie,* **34**, 570-5 (1979).
5 MERCK&Co. Inc., *Neth. Appl.* 6,510,290 (1966).
6 Xin, T., Lu, J., Shen, R., Zhang, Y., *Zhongyou Yiyao Gongye Zazhi,* **21(8)**, 347-50 (1990). Chem. Abstr., **114** 143240m.
7 Perçiner, H., Abbasoglu, A., Noyanalpan, N., *J. Fac. Pharm. Gazi,* **7(2)**, 125-40 (1990).
8 Srivastava, R. S., *Indian Journal of Chemistry,* **29/A**, 1024-26 (1990).
9 Srivastava, R. S., *Inorg. Chim. Acta,* **46**, 43-7 (1980).
10 Kharizanova, T., Torlakov, I., Zhelyazkov, L., Todorova, N., Sheikov, N., *Tr. Nauchnoized. Khim.-Farm. Inst.,* **8**, 347-52 (1972). Torlakov, I., Kharizanova, T., Zhelyazkov, L., Todorova, N., *Tr. Nauchnoized. Khim.-Farm. Inst.,* **9**, 325-31 (1974).
11 Tabata, T., Kondo, T., *Mokuzai Gakkaishi,* **23(10)**, 504-8 (1977). Shinohara, A., Kusano, S., *Japan Kokai,* 78,29,934 (1979).
12 Denisova, L. I., Kosareva, V. M., Lotukhova, K. E., Solonenko, I. G., *Khim-Farm. Zh.,* **9**(9) (1975) 18-21.
13 Piggott, B., Thorpe, S. D., *Inorg. Chim. Acta,* **103(1)**, 3-4 (1985).
14 O'sullivan, D. G., Sadler, P. W., *Nature,* **192**, 341-3 (1961). Chem. Abstr., **61** 6065g.
15 Takuzo, H., Masataka. I., *Chem. Pharm. Bull.,* **30(8)**, 2996-04 (1982).
16 Haugwitz, R. D., Maurer, B. V., Jacobs, G. A., Narayanan, V. L., Cruthers, L., Szanto, J., *J. Med. Chem.,* **22(9)**, 1113-18 (1979).
17 Dol'nikov, Y. Y., Epel'dimov, L. S., *Sb. Rab. Gel'mintol,* **1971**, 129-33.
18 Foks, H., Janowiec, M., *Acta Pol. Pharm.,* **35(3)**, 281-8 (1978).
19 Clark, R. B., Ferreira, S. H., Mehta, N. B., Thorogodd, P. B., Vinegar, R., *Ger. Offen* 2,634,409 (1975).
20 Nose, T., Kono, T., *Jpn. Kokai Tokkyo Koho* 79 95,734 (1979); Chem. Abstr., **92** P82430b.
21 Konovalova, L. M., Ozeretskovskaya, N. N., Kolosova, M. O., *Med. Parazitol. Parazit. Bolez.,* **35(5)**, 551-6 (1966); Chem. Abstr., **66** 84553f.
22 Imperial Chemical Industries of Australia and Nav Zealand Ltd., *Belg.* 631,490 (1963).
23 O'sullivan, D. G., Wallis, A. K., *Nature,* **198**, 1270-3(1963); Sumiyuki, A., Katsuo, S. T., *Meiji Yakka Daigaku Kenkyu Kiyo,* **4**, 25-8 (1974).
24 Çakır, B., Büyükbingöl, E., Uçucu, Ü., Abbasoglu, U., Noyanalpan, N., *Gazi Üniv. Ecz. Fak. Derg.,* **5(1)**, 71-77 (1988).
25 Akihama, S., Takahashi, K., Miyajama, N., *Yakugaku Zasshi,* **94(2)**, 247-51 (1974).
26 Phillips, M. A., *J. Chem. Soc.,* **1928**, 2393; Phillips, M. A., *ibid.,* **1929**, 2820; Shriner, R. L., Upson, R. W., *J. Amer. Chem. Soc.,* **63**, 2277-8 (1941).
27 Ciba Ltd., *Swiss* 240,109 (1946). Granacher, C., Ackerman, F., *U.S.* 2,488,094 (1948); Chem. Abstr., **44** 2250.
28 Ichikawa, M., Nabeya, S., Muraoka, K., Hisano, T., *Chem. Pharm. Bull.,* **27(5)**, 1255-64 (1979).
29 Kwik, W. L., Ang, K. P., *Aust. J. Chem.,* **31**, 459-63 (1978).
30 Kwik, W. L., Ang, K. P., *J. Chem. Soc., Dalton Trans.,* **1981**, 452-55.
31 Chen, X. M., Ye, B. H., Huang, X. C., Xu, Z. T., *J. Chem. Soc., Dalton Trans.,* **16**, 3465-8 (1996).
32 Boswell, J. S., Reedy, B. J., Kulathila, R., Merkler, D., Blackburn, N. J., *Biochemistry,* **35(38)**, 12241-50 (1996).

**Received: April 9, 1999 - Accepted: May 3, 1999 - Received in revised camera-ready format: May 17, 1999**

